# Brain activity associated with pain in inherited erythromelalgia: stimulus-free pain engages brain areas involved in valuation and learning

**DOI:** 10.1016/j.ynpai.2018.01.002

**Published:** 2018-01-31

**Authors:** Paul Geha, Betsy R. Schulman, Sulayman D. Dib-Hajj, Stephen G. Waxman

**Affiliations:** a Department of Psychiatry, Yale School of Medicine, New Haven, CT 06511, United States; b The John B. Pierce Laboratory, New Haven CT 06519, United States; c Department of Neurology, Yale University School of Medicine, New Haven, CT 06510, United States; d Neurorehabilitation Research Center, Veterans Affairs Hospital, West Haven, CT 06516, United States

**Keywords:** Erythromelalgia, Pain, fMRI, Somatosensory, Valuation, Prefrontal

## Abstract

Inherited erythromelalgia (IEM) is a chronic pain disorder caused by gain-of-function mutations of peripheral sodium channel Nav1.7, in which warmth triggers severe pain. Little is known about the brain representation of pain in IEM. Here we study two subjects with the IEM Nav1.7-S241T mutation using functional brain imaging (fMRI). Subjects were scanned during each of five visits. During each scan, pain was first triggered using a warming boot and subjects rated their thermal-heat pain. Next, the thermal stimulus was terminated and subjects rated stimulus-free pain. Last, subjects performed a control visual rating task. Thermal-heat induced pain mapped to the frontal gyrus, ventro-medial prefrontal cortex, superior parietal lobule, supplementary motor area, insula, primary and secondary somato-sensory motor cortices, dorsal and ventral striatum, amygdala, and hippocampus. Stimulus-free pain, by contrast, mapped mainly to the frontal cortex, including dorsal, ventral and medial prefrontal cortex, and supplementary motor area. Examination of time periods when stimulus-free pain was changing showed further activations in the valuation network including the rostral anterior cingulate cortex, striatum and amygdala, in addition to brainstem, thalamus, and insula. We conclude that, similar to other chronic pain conditions, the brain representation of stimulus-free pain during an attack in subjects with IEM engages brain areas involved in acute pain as well as valuation and learning.

## Introduction

Chronic pain is a burden to subjects and society. Subjects suffering from chronic pain have a poor quality of life (Currie and Wang, 2004; Knaster et al., 2012), but there is a paucity of tools to objectively assess pain experience. Functional brain imaging (fMRI) is a valuable tool for investigating brain activity associated with pain (Davis and Moayedi, 2013; Lee and Tracey, 2013; Schmidt-Wilcke, 2015). FMRI has been used to study multiple types of chronic pain, including chronic back pain (Baliki et al., 2006; Baliki et al., 2008b; Ceko et al., 2015; Hashmi et al., 2013; Seminowicz et al., 2011), migraine (Burstein et al., 2015; Schulte and May, 2016), neuropathic pain (Cauda et al., 2010; Cauda et al., 2009; Erpelding et al., 2014; Geha et al., 2007; Geha et al., 2008a; Khan et al., 2014; Maihofner et al., 2003; Malinen et al., 2010), knee osteoarthritis (Parks et al., 2011; Rodriguez-Raecke et al., 2009; Rodriguez-Raecke et al., 2013), fibromyalgia (Flodin et al., 2014; Kuchinad et al., 2007; Loggia et al., 2014; Loggia et al., 2013; Lopez-Sola et al., 2016; Napadow et al., 2010; Schmidt-Wilcke et al., 2014), and chronic pelvic pain (Farmer et al., 2011). These studies have identified structural and functional alterations associated with chronic pain affecting both sensory and limbic brain systems. Importantly, recent evidence suggested that some of these changes may be predictive of the risk of transition from acute to chronic pain (Baliki et al., 2012; Vachon-Presseau et al., 2016). Hence, brain-imaging findings point to brain vulnerabilities to persistence of pain and to brain plasticity in response to pain (Flor et al., 1997; Karl et al., 2001; Maihofner et al., 2007; Maihofner et al., 2003). Nevertheless, the pathophysiology of chronic non-cancer pain in humans remains incompletely understood. One hurdle to reaching this mechanistic understanding is the difficulty of examining how peripheral pathologies from possible tissue injuries interact with brain activity and structure to result in “chronification” of pain.

Inherited eryhthromelalgia (IEM) offers an opportunity to overcome this hurdle and shed some light on the peripheral-central interactions. IEM is a genetic model of neuropathic pain in which severe pain arises from hyperexcitability of peripheral dorsal root ganglion (DRG) neurons (Dib-Hajj et al., 2013). It is characterized by severe burning pain in the distal extremities triggered by mild warmth (Drenth and Waxman, 2007). Gain-of-function mutations in peripheral sodium channel Nav1.7 cause IEM, and thus IEM has a clear molecular basis. The majority of Nav1.7 mutations that cause IEM shift channel activation in a hyperpolarizing direction, making it easier to open the channel; when expressed within DRG neurons, these mutations produce hyper-excitability (Dib-Hajj et al., 2005; Dib-Hajj et al., 2013).

Despite the fact that IEM produces pain with a clear genetic etiology and a well-established basis of peripheral hyperexcitability, little is known about the pattern of brain of activity in subjects suffering from IEM, with only one prior paper describing a single subject (Segerdahl et al., 2012). We have recently completed a fMRI study on the efficacy of the sodium channel blocking drug carbamazepine (Geha et al., 2016) in two subjects with IEM carrying the Na_V_1.7 S241T mutation, which is known to hyperpolarize activation of Nav1.7 (Lampert et al., 2006), and produces profound hyperexcitability in DRG neurons, reducing their threshold and increasing the frequency of their firing (Yang et al., 2012). These subjects had suffered from severe pain for more than a decade due to IEM. Functional MRI data were collected as they reported their pain intensity, during a period of warming which triggered an IEM attack and after termination of the thermal stimulus, the latter allowing the measurement of brain activity associated with pain during an attack in the absence of ongoing external stimulation. Here, we present the brain representation of pain in subjects with IEM, both during exposure to warm stimuli and during the stimulus-free period of pain following cessation of the warmth challenge. We hypothesized that hyper-excitable nociceptors in IEM would activate brain areas usually seen in acute pain such as thalamus, primary sensory/motor areas, insula, and anterior cingulate cortex. In addition, we hypothesize that given the chronic nature of the condition, increased engagement of the brain limbic system would be observed while patients rate their stimulus-free IEM pain.

## Materials and methods

### Participants

The subjects and their clinical pain characteristics were recently reported (Geha et al., 2016). Briefly, the subjects were 2 adults, one male (Subject 1, age = 28 at consent) and his mother (Subject 2, age = 59 at consent) who suffer from IEM with onset of symptoms at the age of 16 and 17, respectively. Subject 1 reported severe burning pain in his feet, triggered by mild warmth, with symptoms progressing to affect his hands, knees, elbows, shoulders and ears, while subject 2 reported progression to affect her knees and ears. Both subjects reported severe pain episodes, which they rated at 8–9 on the numerical rating pain scale (NRS), and subject 1 reported frequent sleep disruption due to this pain. For subject 1, venlafaxine and gabapentin did not provide relief, while lidocaine patches provided minimal relief. Subject 2 took only aspirin for treatment, and reported no relief.

### Study design

The Human Investigations Committees at Yale University and West Haven Veterans Affairs Medical Center approved this study (NCT 02214615). Signed informed consent was obtained from both subjects prior to start of the study. The results reported here were obtained during a double-blind cross-over study where the efficacy of carbamazepine was assessed in each of the two subjects with IEM carrying the S241T mutation of Nav1.7 during a total of seven hospital visits: one pre-scanning/training visit, five scanning visits, and a non-scanning visit for cross-over (visit 5) (Supplementary Table 1). Our observations on the efficacy of carbamazepine in these subjects have been reported separately (Geha et al., 2016). Here we report the brain map for pain in subjects with IEM by collapsing pain rating runs and control visual rating scans across visits (see below)

### Pre-scan testing

IEM subjects are known to have heightened sensitivity to thermal stimuli whereby they experience severe burning pain at mildly warm temperatures that are not experienced as painful in healthy controls (Dib-Hajj et al., 2007). Pain of reproducible intensity in these subjects was evoked using a calibrated warming boot with circulating water maintained at controlled temperatures via a thermal bath. The boot was fitted during all testing to the right foot in both subjects, described by participants as most sensitive to changes in ambient temperature. A thermocouple was attached to the skin under the boot to measure skin temperatures. The temperature threshold to trigger pain was determined during the pre-study visit, with each subject exposed to increasing temperature stimuli starting at 31–32 °C for a duration of 5 min, a duration reported to elicit pain attacks (Segerdahl et al., 2012). If the subject did not report the onset of burning pain similar to an IEM attack after 5 min, the temperature was increased by 2 °C and the stimulus was maintained at the new temperature for 5 min. A reproducible thermal threshold that triggered pain in subject 1 was T = 39.5–40.5 °C, and was T = 37–38 °C for subject 2. These temperatures were used during the initial run in all the subsequent testing sessions to elicit pain attacks. Once a pain attack started we terminated heat stimulation if pain intensity rating reached “very strong” on the generalized labeled magnitude scale (gLMS) (Green et al., 1996) (See below). The ratings are then converted to 0–100 values.

### Continuous pain rating

We collected continuous ratings of pain intensity as described by Foss et al. (2006), and as used previously by the authors (Baliki et al., 2008b; Geha et al., 2007). This method allows the identification of a population-specific brain pain map since it captures stimulus-free fluctuations of chronic pain (Apkarian et al., 2009; Baliki et al., 2006; Geha et al., 2007; Geha et al., 2008b). Subjects indicated continuously their level of pain through a linear potentiometer device attached to the thumb and index finger of the dominant hand, with voltage output collected and calibrated by a computer running LabView software (National instruments, Austin, TX). Subjects were seated in front of a computer monitor, which displayed the extent of their finger span by a colored bar (y axis has an intensity scale of no sensation-worst imaginable sensation), providing visual feedback of their rating. Ratings were sampled at 20 Hz. Subjects were initially trained to use the finger-span device with a moving bar on the computer screen that varied in time, and were instructed to track its length with the finger-span device over a one-minute trial. Both subjects met the criteria of being able to follow the bar at a consistency level that resulted in a correlation coefficient r > 0.75 between rating and bar fluctuations within two attempts. Subjects were then instructed to rate the fluctuations of their own ongoing pain during testing sessions, using the maximum thumb-finger-span to indicate “worst imaginable intensity of pain” and thumb and finger touching to indicate “no pain sensation” on the gLMS. Before testing, each subject was trained to use the general gLMS to rate overall pain intensity (Green et al., 1996). The instruction given to patients while rating their stimulus-free pain or the thermal heat pain was always “Rate the intensity of your pain between no sensation and the worst imaginable pain ever”. The instructions given while rating the magnitude of a moving bar was always “Rate the magnitude of the bar. If the bar is moving up you should increase your finger span; if the bar is moving down you should reduce your finger span. Keep in mind: if the bar is at no sensation your fingers should be touching (i.e. finger span closed). If the bar is at the worst imaginable sensation your fingers should be open to your maximum finger span.”

### Pain rating and visual magnitude rating tasks during fMRI

During each visit subjects were scanned in the following order: (I) while rating their pain in response to thermal stimuli, (II) while rating their ongoing IEM pain (no stimulation) after an attack was elicited and (III) while rating the magnitude of a moving bar using the finger span device (Apkarian et al., 2009). Data was collected between January and May 2015. Details of scans collected at each visit are presented in Supplementary Table 1.

The voltage of the finger-span device was digitized, time-stamped in reference to the fMRI acquisition and connected to a computer providing visual feedback. Every scanning run was 20 min long. The first run was always the thermal stimulation run, which invariably elicited an IEM attack described at session debriefing by both participants to be similar to attacks they experience during daily life. We titrated the thermal stimulation until pain intensity rating reached a pre-determined level of “very strong” on the gLMS during all scanning visits. Thermal runs started with 4 min of no stimulation, followed by two thermal stimuli at temperatures defined during the pre-scanning visit. The thermal stimuli were terminated if subjects indicated pain intensity above “very strong” on the gLMS scale (Green et al., 1996). Hence, patient 1 received one stimulus for 4 min and another one for one minute; patient 2 received 2 stimuli of 4 min duration each.

During each visit, subsequent pain runs were collected without thermal stimulation immediately after the first (thermal stimulation) run. During the latter runs, subjects rated spontaneous fluctuations of their pain. A visual magnitude rating was performed last, as a control to account for visuo-spatial and attention components inherent in our pain rating tasks (Apkarian et al., 2009; Baliki et al., 2008a). The participants’ own pain-rating time series was used as input during the magnitude rating control task. Given the relatively long duration of scanning (~120 min) we repeatedly asked our patients if they needed a rest period to maintain comfort and minimize head motion. When needed, patients were taken out of the scanner for 10–15 min before the study resumed.

### fMRI data acquisition parameters

Imaging data were acquired with a Siemens 3T Trio magnetom scanner at Yale University Magnetic Resonance Research Center. Blood oxygen level dependent (BOLD) images were acquired with the following parameters: voxel resolution = 2 × 2 × 2 mm; TR = 1000 ms; TE = 30 ms; flip angle = 60°; number of volumes = 1200 (20 min); FOV = 220 mm and 60 slices with a multiband acceleration factor = 4. A high-resolution 1 × 1 × 1 mm T1-weighted three-dimensional anatomical image was acquired for each subject with the following parameters: FOV = 250 mm; TR = 1900 ms; TE = 2.52 ms and flip angle = 9°.

### fMRI data analysis

Image analysis was performed on each subject’s data using the Oxford Center for Functional MRI of the brain (FMRIB) Expert Analysis Tool [FEAT (Smith et al., 2004) www.fmrib.ox.ac.uk/fsl]. The preprocessing of each subject’s time-series of fMRI volumes encompassed: skull extraction using Brain Extraction Tool (BET); slice time correction; motion correction; spatial smoothing using a Gaussian kernel of full-width-half-maximum 5 mm; non-linear high-pass temporal filtering (128 s) and subtraction of the mean of each voxel time-course from that time-course. Anatomical and functional images were normalized to the standard Montreal Neurological Institute template brain implemented in FSL. The fMRI signal was linearly modeled on a voxel-by-voxel basis using the FMRIB Improved Linear Model (FILM) with local autocorrelation correction. The six vectors of head motion, their first derivatives, two averaged brain activity signals from the left and right white matter and one from the ventricles were regressed out of the model to correct for head motion and physiologic noise.

After pre-processing, a design matrix was created using the subjects’ continuous pain or visual rating. The general linear model was used to estimate, at each voxel, condition-specific effects. A canonical hemo-dynamic response function consisting of a double-gamma variate function was used to model neural response to events. The significance of the model fit to each voxel time-series was calculated, yielding statistical parametric maps for each subject. All group statistical maps were generated by a second-level fixed effects group analysis, using FMRIB (Flame).

**Fig. 1.**
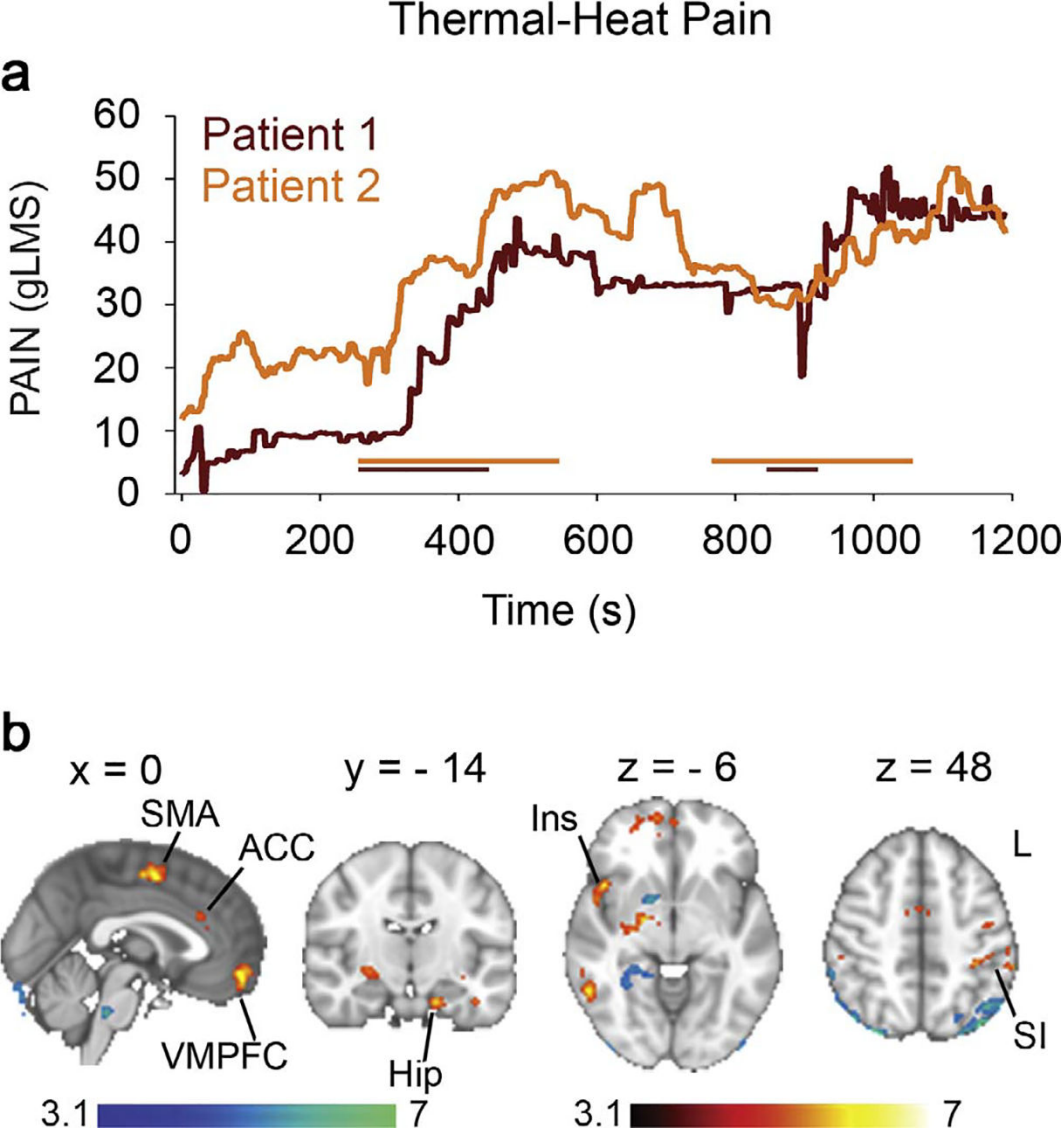
Thermal heat pain. (a) Example of two thermal pain ratings obtained while the warming boot was turned on and off. The colored horizontal lines indicate the timings of the thermal stimuli. (b) Brain activity associated with thermal pain in subjects with IEM due to the S241T Nav1.7 mutation. Brain activity associated with rating of thermal pain in subjects with IEM. Areas shown in red to yellow exhibit significant increase (Z > 3.1, p < 0.05, corrected) in activity during thermal pain rating; areas shown in blue to light blue exhibit significant increase in activity during visual rating *Abbreviations:* R, right; L, Left; ACC, anterior cingulate cortex; Hip, hippocampus; Ins, Insula; SMA, supplementary motor area (SMA); SI, primary somatosensory cortex. Color bars, heat maps for unpaired *t*-test statistics. (For interpretation of the references to color in this figure legend, the reader is referred to the web version of this article.)

**Fig. 2.**
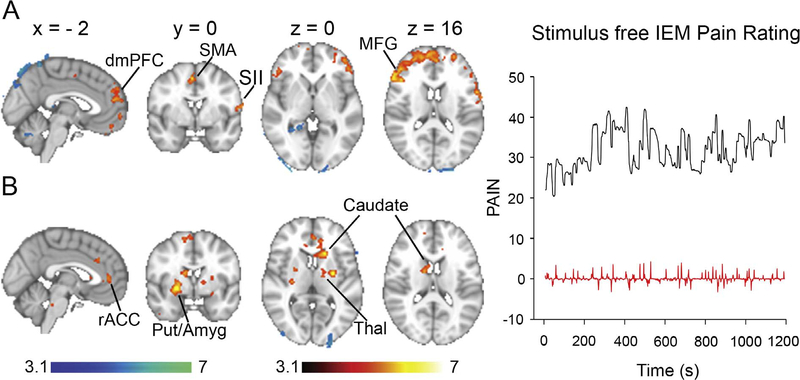
Brain activity associated with stimulus-free pain in patients with IEM due to the S241T Nav1.7 mutation. Areas in red to yellow exhibit significant increase in brain activity during pain rating while areas in blue to light blue exhibit significant increase in brain activity during visual rating (a) Brain activity during stimulus-free pain rating; an example rating is shown on the right in black. (b) Brain activity during periods of change of stimulus-free pain (i.e. derivative of pain rating). An example rating is shown on the right in red. The timeserie in red is the derivative of the pain timeserie depicted in black. *Abbreviations:* Amyg, amygdala; Put, putamen; rACC, rostral anterior cingulate cortex Thal, thalamus. (For interpretation of the references to color in this figure legend, the reader is referred to the web version of this article.)

We first subtracted the visual map from the pain maps to obtain the effect (PAIN minus VISUAL) for each visit for each subject. In the next step, we averaged all visits within subjects and in the final next step we averaged across subjects. The scans included and averaged were collected during pre-treatment baseline, placebo and carbamazepine treatment sessions (i.e. visit). Briefly, first, the contrast of pain minus visual was calculated within each session for thermal heat pain; second, these maps (5 maps for subject 1 and 4 maps for subject 2) were averaged across sessions within each subject; and third, the maps (n = 2) were averaged across the 2 subjects which gives the results depicted in Fig. 1. Hence, the final result is an average map across baseline, placebo and carbamazepine. The same method was followed for stimulus-free pain depicted in Fig. 2.

For each resulting cluster of spatially connected voxels surviving the z threshold of 3.1, a cluster probability threshold of P < 0.05 (family-wise error rate corrected) was applied to the computed significance of that cluster to correct for multiple comparisons (Worsley, 2001). We use a voxel z threshold of 3.1 (corresponding to a p-value < 0.001) given recent recommendations for avoiding false positive activations (Eklund et al., 2016).

## Results

### Thermal heat stimulation in IEM

The two patients reported on average a pain intensity of 32 out of 100 and a maximum pain reaching 52 during thermal heat stimulation at temperatures that would not elicit pain in healthy participants (< 40.5 °C). Pain ratings collected during thermal heat stimulation were used in a general linear model (GLM) analysis to identify the corresponding brain activity (Fig. 1a). Activated brain areas (THERMAL PAIN minus VISUAL; whole brain corrected, Z > 3.1, p < 0.05, corrected) included the right ventral pallidum/amygdala, bilateral primary sensory motor areas (SI/MI), right premotor area, right insula, right putamen, right inferior, middle, and superior frontal gyri, right superior parietal lobule, right temporal cortex, left hippocampus, left secondary somatosensory cortex (SII), left premotor area, left anterior insula/inferior frontal gyrus, left middle frontal gyrus, left inferior parietal lobule, left temporal cortex, in addition to supplementary motor area (SMA), anterior cingulate cortex (ACC) (Brodmann Area (BA) 32), and ventro-medial prefrontal cortex (VMPFC) (Fig. 1b red to yellow; Table 1).

The opposite contrast (VISUAL minus THERMAL PAIN) showed increased activity in the brainstem, cerebellum, left temporal cortex and right ventral pallidum (Fig. 1, blue to light blue). We inspected the response to thermal heat at a lower threshold (Z > 2.3, p < 0.05, corrected) to see whether we could observe activations in the thalamus or primary sensory/motor foot area. Surprisingly, thalamic activations were absent but we identified mid-line sensory/motor activations in the foot area (Supplementary Fig. 1).

### Stimulus-free pain

Both patients reported low to nil pain scores varying between 0 and 3 on the NRS with an average of ~1 upon presentation for the scanning sessions. Pain was triggered after they received a thermal stimulus, which was terminated after 1–5 min, and tended to fluctuate (Fig. 2, black trace).

The average pain intensity was 35. The fluctuations in stimulus-free pain were used in a GLM to identify the corresponding brain activity. Pain (corrected for visual ratings) correlated to activity mainly in the dorso-medial and ventro-medial prefrontal cortex, in addition to SMA and left frontal operculum/SI (Fig. 2a red to yellow, Table 2). The opposite contrast yielded significant activations in the cerebellum and the visual cortex (Fig. 2a blue to light blue, Table 2). Examination of brain activity during episodes of change in stimulus-free pain (Fig. 2, red trace) showed significant activations in the subjective valuation network (Kable and Glimcher, 2007) limbic system (Mesulam, 2000) including rostral ACC, dorsal and ventral striatum (caudate and putamen), bilateral amygdala, in addition to SMA and left thalamus (Fig. 2b, Table 3). The qualitative differences observed between thermal heat pain depicted in Fig. 1b and stimulus-free pain depicted in Fig. 2 could have been due to differences in the experienced pain intensity. Therefore, we calculated the average pain rating intensities per condition at each visit (Supplementary Table 2) and compared them between thermal heat pain and stimulus-free pain using paired *t*-test. The average thermal heat pain rating (± standard error of the mean) across all visits was 31.1 ± 3.1 and the average stimulus-free pain was 35.6 ± 5.1. The difference was not significant (p = 0.41).

## Discussion

Our results show that, similar to acute and chronic pain studies, IEM pain activates both sensory/motor and limbic areas (Apkarian et al., 2005; Schmidt-Wilcke, 2015). Acute thermal pain in IEM engaged areas often collectively referred to as the “pain matrix” (Tracey and Mantyh, 2007) including primary and secondary somatosensory motor areas (SI/SII, MI/MII and SMA), insula, and ACC, in addition to areas of the limbic system (Mesulam, 2000) including hippocampus, amygdala and VMPFC. Pain in IEM is caused by abnormal impulse activity in peripheral sensory DRG neurons including nociceptors in which gain-of-function mutations of the Na_V_1.7 sodium channel produce hyper-excitability (Dib-Hajj et al., 2013; Drenth and Waxman, 2007; Namer et al., 2015). Activation of these primary sensory neurons with noxious stimuli in healthy participants during acute-pain very commonly leads to activation of the thalamus, primary and secondary somatosensory areas, insula and ACC (Apkarian et al., 2005; Jensen et al., 2016; Tracey and Mantyh, 2007; Wager et al., 2013). In contrast, stimulus-free IEM pain engaged mainly fronto-striatal limbic circuits involved in subjective valuation (rostral ACC, striatum) (Hart et al., 2014; Kable and Glimcher, 2007; Levy and Glimcher, 2012), emotional processing (LeDoux, 2000; Phelps and LeDoux, 2005), and decision-making (striatum, hippocampus, amygdala, dorsal and medial PFC) (Grabenhorst and Rolls, 2011).

It is notable that although the same peripheral fibers are engaged in stimulus-free and acute thermal IEM pain, and despite comparable levels of pain intensity, stimulus-free IEM pain engaged mainly the fronto-striatal limbic system. Our results are consistent with the observation that repetitive acute pain stimulation over several days in healthy participants leads to decreased activation in the areas of the “pain matrix” and increased activity in the sub-genual ACC (Bingel et al., 2007). Persistent firing of peripheral pain fibers could have occurred after we terminated the thermal stimulus in our experiments, hence driving pain perception via long-term potentiation (Sandkuhler, 2007). Persistent afferent firing after termination of a thermal stimulus triggering an IEM attack mimics situations that our patients encounter in everyday life where transient exposure to higher temperature triggers pain attacks that outlast the exposure to warmth. Together, these observations indicate that, despite similar peripheral nociceptive input, the brain representation of acute pain during thermal stimulation might be distinct from the pattern associated with pain that outlasts warmth in IEM. This may possibly be due to the neuroplastic changes including learning that accompany chronic pain (Dellarole et al., 2014; Dimitrov et al., 2014; Duric and McCarson, 2006; Mutso et al., 2012). Hence, a sustained barrage of peripheral nociceptive input would lead to cortical and sub-cortical neuroplastic changes (Johansen et al., 2011), which could in turn integrate noxious input into a negative motivational state (Borsook et al., 2016). This hypothesis is consistent with the observed increased activations of areas of the limbic system like amygdala, hippocampus and VMPFC and striatal areas, which are all well known to mediate learning (Balleine and O’Doherty, 2010; Hart et al., 2014).

Segerdahl et al. (2012) reported brain activity using arterial spin labeling in one patient with IEM by comparing periods of painful attacks to periods of pain relief; pain relief lead to a significant decrease in activity of inferior frontal gyrus, VMPFC, insula, ACC, SI, thalamus, and dorsal striatum. The differences between the two experimental setups preclude direct comparison between the two studies. Nevertheless, we observed overlaps with the findings of Segherdahl et al. in the striatum, insula, ACC and VMPFC. The absence of significant thalamic activation during thermal heat stimulation in our data is intriguing although we did observe significant activations in both SI and SII and thalamic activation when stimulus-free pain was changing. One explanation is that our visual control task activates the thalamus as well (Baliki et al., 2009; Baliki et al., 2008a); therefore, contrasting pain ratings with visual rating and averaging across a small number of subjects might not have enough power to detect such activations.

Our approach captures stimulus-free pain in IEM; it is novel in that it allows the recording of the subjective experience of a tonic IEM attack without the sensory input from an outside salient stimulus. Activations observed during acute pain in healthy subjects engaging the “acute pain matrix” are sufficient to predict pain (Wager et al., 2013). In addition, some fMRI studies of chronic pain have reported decreased activations in the thalamus (Di Piero et al., 1991; Duncan et al., 1998; Fukumoto et al., 1999; Hsieh et al., 1996; Iadarola et al., 1995; Mountz et al., 1995), and abnormal activations and functional connectivity in the insula (Ceko et al., 2015; Hong et al., 2014; Lopez-Sola et al., 2017; Malinen et al., 2010; Napadow et al., 2012; Napadow et al., 2010) and ACC (Loggia et al., 2014; Lopez-Sola et al., 2017; Malinen et al., 2010). Nevertheless, the specificity of the activations in areas of the “pain matrix” has been questioned since they can be elicited by other salient sensory stimuli (Legrain et al., 2011; Mouraux et al., 2011) and are observed in patients with congenital insensitivity to pain (Salomons et al., 2016). Furthermore, activity in the visual cortex, which does not receive direct nociceptive input, can be sufficient to predict acute pain (Liang et al., 2013). The non-specificity of activations elicited by acute pain is consistent with stimulation studies in humans showing that a very small fraction of neuronal stimulations in the posterior insula/SII only are capable of eliciting pain (Mazzola et al., 2012). By contrast, stimulus-free pain rating has been helpful in identifying population specific brain components of chronic pain in other conditions (Apkarian et al., 2009). For example, stimulus-free pain ratings have been consistently shown to correlate with brain activity in the nucleus accumbens, amygdala and rostral anterior cingulate/medial prefrontal cortex (Baliki et al., 2006; Geha et al., 2007; Hashmi et al., 2013). Taken together, these results suggest that stimulus-free pain may help uncover subjective aspects of chronic pain which are repeatedly observed to relate more to activity in areas of the limbic brain (Mesulam, 2000) and to relate less to areas involved in salience detection and sensory processing (Legrain et al., 2011).

In our previous study (Geha et al., 2016) we used fMRI to assess the effect on pain of carbamazepine compared to placebo in the subjects described here. Genomic analysis, structural modeling, and in vitro functional assessment pointing to a specific effect of carbamazepine on the mutated Nav 1.7 channel carried by both subjects guided treatment with carbamazepine. Clinical pain improvement was observed with carbamazepine but not placebo, and was accompanied by a decrease in activity of valuation areas, mainly VS, ACC and posterior cingulate cortex (Kable and Glimcher, 2007; Levy and Glimcher, 2012). These same areas were observed when we examined time periods when stimulus-free IEM pain was changing. This suggests that these valuation areas are good targets for therapy. Recent data from rodent models support an important role for the ventral striatum-VMPFC circuitry in chronic pain. This circuitry has been reported to access peripheral nociceptive input via direct projections from the spinal cord (Braz et al., 2005; Gauriau and Bernard, 2002; Han et al., 2015; Ma and Peschanski, 1988; Newman et al., 1996), and indirect projections from the brainstem, thalamus, limbic brain, and insula (Haber and Knutson, 2010). Lee et al. demonstrated that optogenetic activation of prelimbic projections from PFC, the equivalent of VMPFC in rodents, to the VS, i.e. nucleus accumbens, results in a decrease in sensory and affective pain behavior (Lee et al., 2015). Ren et al. showed that altering the excitability of accumbens spiny projection neurons modulates nociceptive behavior in rats, providing further evidence that VS can gate ascending nociceptive activity (Ren et al., 2016). Taken together, results of these animal studies and the prior (Geha et al., 2016) and present human studies support the important role for the cortico-striatal circuitry in representation of pain in subjects with IEM, and suggest it as a potential anatomic target for intervention to modulate and treat IEM as has been suggested for other idiopathic chronic pain conditions (Baliki and Apkarian, 2015).

## Conclusion

In summary, our observations in two subjects with the S241T Nav1.7 mutation and IEM demonstrate an overlap between IEM and other chronic clinical pain conditions. We suggest that the sustained barrage of peripheral sensory input in different chronic pain conditions leads to a reorganization in the brain’s representation of pain and increased involvement of the brain limbic system.

## Supplementary Material

1

## Figures and Tables

**Table 1:** Activation during thermal heat stimulation.

Regions	*Coordinates (mm)*			Z−value	Cluster
	*X*	*Y*	*Z*		
*Contrast 1: Stimulus-Free Pain > Visual*					
R Pre-Motor area (6)	32	−8	68	5.06	1
R SPL	28	−56	64	4.71	2
L Insula/Putamen	−34	−10	−8	4.14.	3
L Pre-Motor area (6)	−36	−8	62	4.75	4
L Hippocampus	−16	−14	−26	4.75	5
R VP/Amygdala	20	−8	−6	4.89	6
L Temporal Pole	−42	6	−23	4.41	7
L Ant Insula/IFG	−26	12	−28	4.75	8
ACC (32)	4	24	28	4.3	9
L SII	−64	−24	22	4.1	10
R MFG	34	44	20	4.75	11
R SFG	26	58	−10	4.39	12
R SI	40	−38	62	5.24	13
L IPL	−58	−38	46	4.75	14
R SFG	22	64	8	5.25	15
R IFG	52	32	12	3.76	16
SMA (6)	2	−2	52	5.66	17
L MFG	−54	38	12	5.58	18
R Temporal Pole	44	22	−32	5.34	19
R Insula	46	14	−6	4.97	20
VMPFC	0	54	−12	5.38	21
R MTG	56	−54	−8	5.21	22
*Contrast 2: Visual > Stimulus-Free Pain*					
R VP	14	6	−8	5.22	1
L ITG	−46	−2	−40	3.82	2
Pons/Cerebellum	−12	−34	−28	4.59	3
R Fusiform G	30	−42	2	5.34	4
Pons	4	−34	−36	5.03	5

Contrast 1 contained 22 clusters: cluster 1, 75 voxels, p < 0.05; cluster 2, 75 voxels, p < 0.05; cluster 3, 81 voxels, p < 0.05; cluster 4, 87 voxels, p < 0.05; cluster 5, 95 voxels, p < 0.05; cluster 6, 108 voxels, p < 0.01; cluster 7, 120 voxel, p < 0.01; cluster 8, 130 voxels, p < 0.01; cluster 9, 132 voxels, p < 0.01; cluster 10, 136 voxels, p < 0.01; cluster 11, 143 voxels, p < 10^−3^; cluster 12, 146 voxels, p < 10^−3^; cluster 13, 159 voxels, p < 10^−3^; cluster 14, 163 voxels, p < 10^−3^; cluster 15, 166 voxels, p < 10^−3^; cluster 16, 167 voxels, p < 10^−3^; cluster 17, 200 voxels, p < 10^−4^; cluster 18, 208 voxels, p < 10^−4^; cluster 19, 242 voxels, p < 10^−4^; cluster 20, 274 voxels, p < 10^−5^; cluster 21, 352 voxels, p < 10^−6^; cluster 22, 367 voxels, p < 10^−6^; Contrast 2 contained 6 clusters: cluster 1, 71 voxels, p < 0.05; cluster 2, 77 voxels, p < 0.05; cluster 3, 145 voxels, p < 10^−3^; cluster 4, 195 voxels, p < 10^−4^; cluster 5, 206 voxels, p < 10^−4^.

Abbreviations: Ant, anterior; ACC, anterior cingulate cortex; IFG, inferior frontal gyrus; MFG, middle frontal gyrus; MTG, middle temporal gyrus; SII, secondary somatosensory cortex; SFG, superior frontal gyrus; SI, primary somatosensory cortex; SMA, supplementary motor area; SPL, superior parietal lobule, VMPFC, ventro-medial prefrontal cortex; VP, ventral putamen.

**Table 2:** Activations associated with stimulus-free IEM pain.

Regions	*Coordinates (mm)*			Z-value	Cluster
	*X*	*Y*	*Z*		
*Contrast 1: Stimulus-Free Pain > Visual*					
SMA (6)	4	−2	54	5	1
OFC (11)	−12	42	−20	4.93	2
LMFG	−20	18	42	4.88	3
R SFG	32	34	50	4.29	4
VMPFC	0	60	−4	4.1	5
L SFG (10)	−34	60	8	5.53	6
R MFG (10)	28	54	14	5.11	6
R SFG (9)	12	62	28	4.8	6
L Fontal Operc./SI	−58	8	16	4.6	6
*Contrast 2: Visual > Stimulus-Free Pain*					
R LG (18)	18	−46	−2	4.75	1
L Cerebellum	−18	−80	−40	4.31	2

Contrast 1 contained 7 clusters: cluster 1, 80 voxels, p < 0.05; cluster 2, 93 voxels, p < 0.05; cluster 3, 120 voxels, p < 0.01; cluster4, 161 voxels, p < 10–3; cluster 5, 195 voxels, p < 10–4; cluster 6, 205 voxels, p < 10–4; cluster 7, 3506 voxels, p < 10–37. Contrast 2 contained 2 clusters: cluster 1, 91 voxels, p < 0.05; cluster 2, 143 voxels, p < 10–3.

Abbreviation: LG, lingual gyrus; Operc, Operculum; OFC, orbitofrontal cortex.

**Table 3:** Activations associated with changing stimulus-free IEM pain.

Regions	*Coordinates (mm)*			Z-value	Cluster
	*X*	*Y*	*Z*		
*Stimulus-Free Derivative of Pain > Derivative of Visual*					
R Caudate	12	0	18	4.4	1
R Cerebellum	8	−60	−32	4.21	2
R Temporal Pole	36	6	−35	3.93	3
L Pons	−10	−30	−30	3.92	4
R SFG (8)	14	54	38	4.37	5
L Putamen	−28	−6	6	5.37	6
L Thalamus	−12	−10	6	3.46	6
rACC (32)	−2	44	6	4.07	7
R Putamen/Amygdala	24	0	−4	5.65	8
R SMA (6)	12	−12	68	4.69	9
L Caudate	−16	22	6	5.06	10
L Insula	−32	10	−10	3.54	10
ACC (24)	16	8	40	5.92	11

Contrast 1 contained 7 clusters: cluster 1, 72 voxels, p < 0.05; cluster 2, 79 voxels, p < 0.05; cluster 3, 86 voxels, p < 0.02; cluster 4, 110 voxels, p < 10–2; cluster 5, 127 voxels, p < 10^−2^; cluster 6, 168 voxels, p < 10^−3^; cluster 7, 170 voxels, p < 10^−3^; cluster 8, 300 voxels, p < 10^−6^; cluster 9, 327 voxels; cluster 10, 369 voxels, 10^−7^; cluster 11, 563 voxels, 10^−10^.

## Abbreviations:

fMRI: functional brain imaging
Ant: anterior
ACC: anterior cingulate cortex
IEM: inherited eryhthromelalgia
IFG: inferior frontal gyrus
MFG: middle frontal gyrus
LG: lingual gyrus
MTG: middle temporal gyrus
OFC: orbitofrontal cortex
Operc: operculum
SII: secondary somatosensory cortex
SFG: superior frontal gyrus
SI: primary somatosensory cortex
SMA: supplementary motor area
SPL: superior parietal lobule
VMPFC: ventro-medial prefrontal cortex
VP: ventral putamen
