# Supplementary material for: Brain activity associated with pain in inherited erythromelalgia: stimulus-free pain engages brain areas involved in valuation and learning
Source: Neurobiol Pain. 2018 Jan 31;3:8–14. doi: 10.1016/j.ynpai.2018.01.002 (PMC6505710; doi:10.1016/j.ynpai.2018.01.002)
Supplement: Supplementary data 1 [file mmc1.docx]

**Supplementary Material**


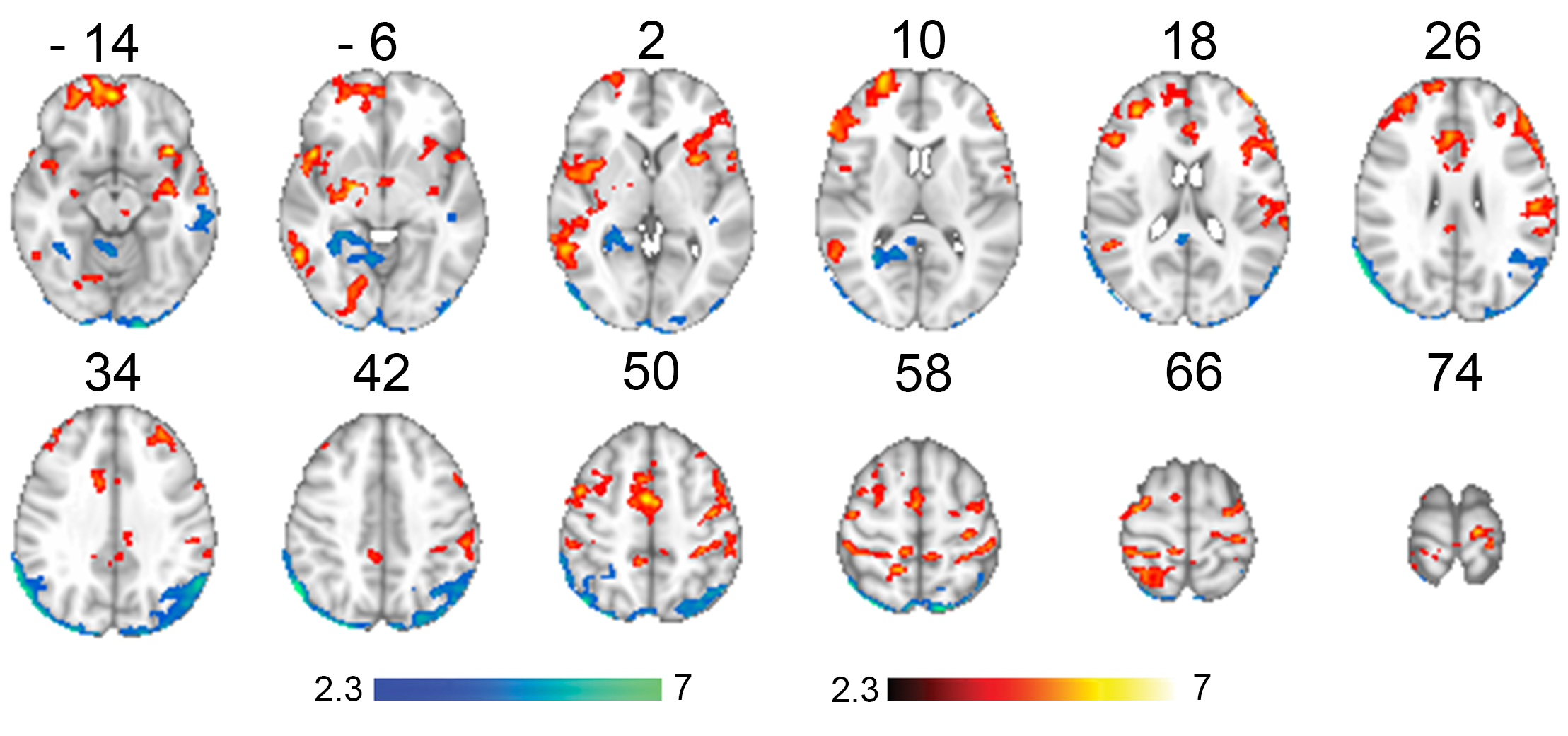


**Supplementary Fig. 1**. Brain activity associated with thermal pain in subjects with IEM due to the S241T Nav1.7 mutation presented at a lower threshold than Figure 1 (Z > 2.3, p < 0.05, corrected). Areas shown in red to yellow exhibit significant increase in activity during thermal pain rating; areas shown in blue to light blue exhibit significant increase in activity during visual rating. No thalamic activity could be identified; however, we do see bilateral activation of the primary somatosensory/motor cortices. *Numbers on top indicate the z-coordinate in mm of standard space.*

**Supplementary Table 1.** Detailed summary of fMRI data collection per subject

|  | **Patient 1** | | | **Patient 2** | | |
| --- | --- | --- | --- | --- | --- | --- |
|  | Thermal | Spontaneous | Visual | Thermal | Spontaneous | Visual |
| **Session 1** | 1 | 2 | 1 | 1 | 1 | 1 |
| **Session 2** | 1 | 2 | 1 | 1 | 1 | 1 |
| **Session 3** | 1 | 2 | 1 | 1 | 2 | 1 |
| **Session 4** | 1 | 2 | 1 | * | * | * |
| **Session 5** | 1 | 1 | 1 | 1 | 2 | 1 |
| Numbers indicate the number of runs per type of scanning; run duration was 20 minutes. *, patient was unable to come. | | | | | | |

**Supplementary Table 2. Average pain intensity ratings at each visit for thermal heat and stimulus-free pain**

|  | Session Type | Thermal heat | Stimulus free |
| --- | --- | --- | --- |
| Patient 1 | Baeseline | 32.6 | 37 |
| Patient 1 | Acute CBZ | 12.2 | 15.9 |
| Patient 1 | Chronic CBZ | 29.4 | 40.7 |
| Patient 1 | Acute placebo | 23 | 40.9 |
| Patient 1 | Chronic placebo | 30.2 | 63.5 |
| Patient 2 | Baseline | 43.3 | 47 |
| Patient 2 | Acute CBZ | * | * |
| Patient 2 | Chronic CBZ | 39.2 | 32.9 |
| Patient 2 | Acute placebo | 35.6 | 15 |
| Patient 2 | Chronic placebo | 34.3 | 27.6 |
|  | Average | 31.1 | 35.6 |
